# Immigrants’ use of emergency primary health care in Norway: a registry-based observational study

**DOI:** 10.1186/1472-6963-12-308

**Published:** 2012-09-07

**Authors:** Hogne Sandvik, Steinar Hunskaar, Esperanza Diaz

**Affiliations:** 1 National Centre for Emergency Primary Health Care, Uni Health, Uni Research, Kalfarveien 31, 5018, Bergen, Norway; 2 Research Group for General Practice, Department of Public Health and Primary Health Care University of Bergen, Kalfarveien 31, 5018, Bergen, Norway

**Keywords:** Norway, Immigrants, Primary health care, Out of hours medical care, Emergency care

## Abstract

**Background:**

Emigrants are often a selected sample and in good health, but migration can have deleterious effects on health. Many immigrant groups report poor health and increased use of health services, and it is often claimed that they tend to use emergency primary health care (EPHC) services for non-urgent purposes. The aim of the present study was to analyse immigrants’ use of EPHC, and to analyse variations according to country of origin, reason for immigration, and length of stay in Norway.

**Methods:**

We conducted a registry based study of all immigrants to Norway, and a subsample of immigrants from Poland, Germany, Iraq and Somalia, and compared them with native Norwegians. The material comprised all electronic compensation claims for EPHC in Norway during 2008. We calculated total contact rates, contact rates for selected diagnostic groups and for services given during consultations. Adjustments for a series of socio-demographic and socio-economic variables were done by multiple logistic regression analyses.

**Results:**

Immigrants as a whole had a lower contact rate than native Norwegians (23.7% versus 27.4%). Total contact rates for Polish and German immigrants (mostly work immigrants) were 11.9% and 7.0%, but for Somalis and Iraqis (mostly asylum seekers) 31.8% and 33.6%. Half of all contacts for Somalis and Iraqis were for non-specific pain, and they had relatively more of their contacts during night than other groups. Immigrants’ rates of psychiatric diagnoses were low, but increased with length of stay in Norway. Work immigrants suffered less from respiratory and gastrointestinal infections, but had more injuries and higher need for sickness certification. All immigrant groups, except Germans, were more often given a sickness certificate than native Norwegians. Use of interpreter was reduced with increasing length of stay. All immigrant groups had an increased need for long consultations, while laboratory tests were most often used for Somalis and Iraqis.

**Conclusions:**

Immigrants use EPHC services less than native Norwegians, but there are large variations among immigrant groups. Work immigrants from Germany and Poland use EPHC considerably less, while asylum seekers from Somalia and Iraq use these services more than native Norwegians.

## Background

The primary health care in Norway is based on a list system with regular general practitioners (RGPs) who act as gate-keepers for secondary care. During office hours most patients with urgent needs consult their RGP, at other times they use the out-of-hours emergency primary health care (EPHC) services. In some cities and municipalities there are also day-time EPHC services. No referral is needed for consulting the EPHC. There is no consultation fee for patients under 16 years of age.

In recent years the inflow of immigrants to Norway has reached record levels. In 2010 the immigration rate was 15 per thousand inhabitants, with 64% from EU countries [1]. At the beginning of 2011 all immigrants and their children constituted 12% of the population, Africans 2% and Asians 4%.

Migration is often a stressful event which can have deleterious effects on health. However, emigrants are a selected sample, and a healthy migrant effect has been described, meaning that newly arrived immigrants are healthier than the average [2,3]. With time the healthy migrant effect may wear off [4,5], and many immigrants report poor health and increased use of health services [6-12]. An unhealthy remigration effect has also been described, often characterized as “salmon bias effect”, meaning that disadvantaged immigrants may remigrate to their home country, e.g. because of health problems [13].

Poor health can be attributed to difficult and dangerous conditions in the country of birth and a stressful migration process, but also by conditions in the host country. Low socio-economic status, poor acculturation, and discrimination seem to be important explanatory factors for immigrants’ poorer health [7-10,12,14]. Employment rates among EU immigrants to Norway are over 70%, while immigrants from Asia have an employment rate of 53% and those from Africa only 44% [1]. The opposite pattern is found for sickness certification and disability pensioning [15,16], mostly explained by work factors and level of income, but also by mental distress and poor health [16].

It has been reported that many immigrant groups tend to use emergency rooms and out-of-hours services for non-urgent purposes [17-19]. Poor knowledge of the health care system, inability to make appointments by phone (language barriers), lack of a regular general practitioner (RGP), and dissatisfaction with the RGP may contribute to increased use of EPHC services [17,19,20]. Inappropriate use of EPHC services will, however, affect the quality of services given. It is often impossible to arrange for an interpreter to be present at the consultation, or to arrange adequate follow-up. Probably, many of these consultations would be better taken care of by a RGP.

The purpose of the present study was to analyse immigrants’ use of EPHC, compared with native Norwegians. At first we included all immigrants, then categorized them by world regions, and finally analysed single countries in more detail. By this strategy, we avoided the common problem of ‘ethnic lumping’ [21]. Most immigrants come to Norway for work or for protection (asylum seekers). To reflect this situation, we selected two typical countries that supply work immigrants (Poland and Germany) and two typical countries that supply asylum seekers (Somalia and Iraq) for further analysis, i.e. use of EPHC according to country of origin, reason for immigration, and length of stay in Norway. By linking high quality national records we obtained a large and complete material, including a series of possibly confounding socio-demographic and socio-economic variables.

## Methods

The material in this study is based on electronic compensation claims for EPHC contacts in Norway during 2008. Contacts with RGPs during office hours are not included. Nearly all claims are electronic, only about 2% are paper based and not included in this material [22].

In Norway the local municipalities are responsible for the EPHC for their inhabitants and visitors, both during office hours and out-of-hours. The organization of the emergency services may differ between municipalities, but all send electronic compensation claims for all patient contacts to the Norwegian Health Economics Administration (HELFO). Thus, HELFO has complete records of all patient contacts with the EPHC. The following HELFO variables were used in this study: Centrality of the municipality, patients’ gender, age, time of contact, diagnosis (ICPC-2, International Classification of Primary Care), and a number of different fee codes.

The centrality is defined as a municipality’s geographical location in relation to a centre where there are important functions (central functions) and is measured on a scale of 0–3 where 0 is the least and 3 is the most central [23].

We categorized some selected ICPC-2 codes into five diagnostic groups: non-specific pain with no diagnosed cause (A11, D01-02, D06, L02-03, N01), injury to musculoskeletal system, head, or skin (L72-81, N79-80, S14-19), psychiatric illness (all P-diagnoses), respiratory infections (R72, R74-78, R80-81, R83), and gastroenteritis (D70, D73).

There are different fee codes for different types of contact and for numerous different procedures. A time fee is claimed when the consultation lasts more than 20 minutes. A specific fee is used when an interpreter is present at the consultation. The time fee and the interpreter fee are mutually exclusive, and cannot be used for the same consultation. There are also specific fees for taking laboratory tests and for writing sickness certificates.

All Norwegian citizens are given a unique personal identification number (ID-number) at birth. This number is used in various official records, including HELFO, and allows for linking such records on an individual level. Foreigners moving to Norway to stay for more than six months are also given an ID-number. A dummy number (D-number) may be issued to foreign nationals staying in Norway for less than six months.

As a rule all medical services will register a patient’s ID-number or D-number. However, in emergency settings these numbers are not always available. The patient may not remember his number or his children’s numbers, or he has no number at all. This is the case for all foreign tourists, some asylum seekers, and persons living illegally in Norway. Because of the need for linking to other records only patients with ID-number were included in this study.

The ID-number made it possible to identify patients who were enlisted with a RGP. Furthermore, Statistics Norway supplied the following variables (also based on the ID-number): Immigrant status, country of origin, reason for immigration, length of stay in Norway (years), citizenship, and annual work income (NOK).

An immigrant was defined as an individual who is born abroad by two foreign parents, and who has since moved to Norway (previously called first generation immigrant), or as an individual who is born in Norway by two immigrant parents (previously called second generation immigrant). A native Norwegian was defined as an individual who is born in Norway by two Norwegian parents. Statistics Norway has recorded reason for immigration since 1990, categorized as protection, work, family reunion, education, other, and unknown. Length of stay is calculated from the time the immigrant is granted a work/residence permit.

Statistics Norway also supplied total population numbers for native Norwegians and immigrants of different origin, and for different age groups, thus enabling us to calculate contact rates for all subgroups. The age distributions of native Norwegians and immigrants are different, and the contact rates were therefore age adjusted. The following areas of origin, a standard set by Statistics Norway, were used:

1. Norway

2. Nordic countries

3. Western Europe

4. Eastern Europe

5. Asia (including Turkey), Africa, Latin America

6. North America, Oceania

In the study of single countries we used multiple logistic regression analyses to explore why an individual patient had or had not received a specific diagnosis or service (dependent variable). Explanatory variables were country of origin (Norway as reference category), reason for immigration (family reunion as reference category), and length of stay (11–18 years as reference category). Immigrant cases were included in the analyses if length of stay was 18 years or less, and if reason for immigration was recorded as protection, work, or family reunion. The multivariate analyses were adjusted for age, gender, centrality, Norwegian citizenship, work income, enlistment with a RGP, and number of EPHC contacts. Significance was accepted at the 5% level (p < 0.05), and odds ratios are presented with 95% confidence interval.

The study is part of the project “Immigrants’ health in Norway” located at the Research Group for General Practice at the Department of Public Health and Primary Health Care, University of Bergen. The project has been approved by the Norwegian Data Inspectorate, the Regional committee for medical research ethics, the Norwegian Labour and Welfare Service, and the Norwegian Directorate of Health. Linking of records was performed by the Norwegian Prescription Database and the Norwegian Social Science Data Service who finally supplied us with the anonymous data file.

## Results

The total number of EPHC contacts during 2008 was 1 715 278. Of these 23.1% lacked ID-number (pseudonym). There were more children < 10 years among those without ID-number than among the rest (38.5%, 95% CI 38.3% - 38.7% versus 15.6%, 15.5% - 15.7%).

**Figure 1:**
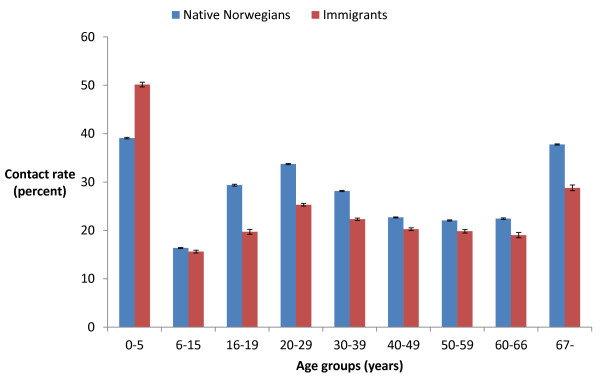
**Yearly EPHC contact rate (95% CI).** All types of contacts, native Norwegians and immigrants, by different age groups (years).

**Figure 2:**
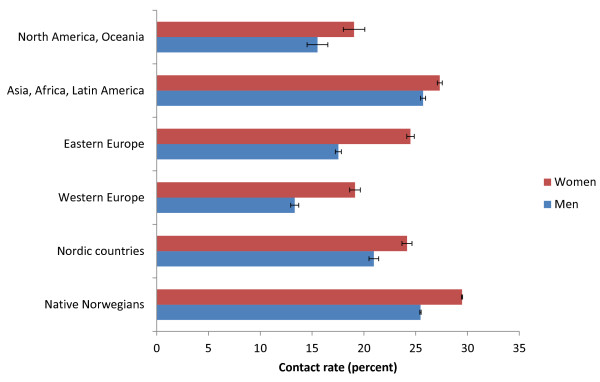
**Age adjusted yearly EPHC contact rate (95% CI).** All types of contacts, native Norwegians and immigrants from different parts of the world.

Immigrants as a whole had a lower contact rate (23.7%, 23.6% - 23.8%) than native Norwegians (27.4%, 27.4% - 27.5%). This trend was similar in all age groups, except for the youngest children (Figure 1). The contact rates varied according to immigrants’ area of origin, but immigrants from all areas had lower contact rates than Norwegians, except men from Asia, Africa and Latin America. Women had higher contact rates than men in all groups (Figure 2).

Of the four immigrant nationalities examined, Iraqis constituted the largest group, Germans the smallest (Table 1). Somalis were youngest, while Poles were mostly males. Germans had the longest stay in Norway and the largest percentage living in rural areas. More than 50% of Somalis and Iraqis had acquired Norwegian citizenship, in contrast to only 15 - 20% of Poles and Germans. More than 40% of Iraqis and Somalis came to Norway for protection, while 48% of Germans and 59% of Poles came to work. In addition, 26 - 33% from all countries came as a result of family reunion. Fewer Poles and Germans were enlisted with a RGP than the other groups.

**Table 1:** Description of the material, native Norwegians and immigrants from Poland, Germany, Somalia, and Iraq (95% CI)

	**Norway**	**Poland**	**Germany**	**Somalia**	**Iraq**
**Number of patients**	684 978	3 658	1 770	4 793	5 382
**Mean age (years)**	40.2 (40.1 – 40.2)	29.5 (29.0 – 30.0)	35.4 (34.4 – 36.4)	21.1 (20.6 – 21.5)	24.9 (24.4 – 25.3)
**Percentage women**	53.4 (53.3 – 53.5)	39.5 (37.9 – 41.1)	53.2 (50.9 – 55.5)	51.7 (50.3 – 53.1)	47.0 (45.7 – 48.3)
**Immigrants’ length of stay in Norway (years)**	-	4.9 (4.6 – 5.2)	10.7 (10.0 – 11.4)	6.2 (6.1 – 6.3)	6.8 (6.7 – 6.9)
**Percentage with Norwegian citizenship**	100.0 (100.0 – 100.0)	15.4 (14.3 – 16.6)	17.4 (15.7 – 19.2)	53.1 (51.7 – 54.5)	53.1 (51.8 – 54.4)
**Percentage rural (centrality 0)**	13.4 (13.3 – 13.5)	5.2 (4.5 – 5.9)	9.9 (8.5 – 11.3)	3.7 (3.2 – 4.2)	2.8 (2.4 – 3.2)
**Percentage enlisted with a RGP**	97.8 (97.8 – 97.8)	92.3 (91.4 – 93.1)	93.3 (92.1 – 94.4)	97.9 (97.4 – 98.3)	98.7 (98.3 – 99.0)
***Reason for immigration*** ***(percentage of all immigrants with ≤ 18 years length of stay):***					
**N**		3 306	1 426	4 638	5 261
**Protection**	-	0.5 (0.3 – 0.7)	1.1 (0.6 – 1.6)	40.6 (39.2 – 42.0)	43.7 (42.4 – 45.0)
**Work**	-	58.7 (57.0 – 60.4)	47.9 (45.3 – 50.5)	0.0 (0.0 – 0.0)	0.2 (0.1 – 0.3)
**Family reunion**	-	29.9 (28.3 – 31.5)	32.9 (30.5 – 35.3)	25.9 (24.6 – 27.2)	30.6 (29.4 – 31.9)
**Other, unknown**	-	10.9 (9.8 – 12.0)	18.1 (16.1 – 20.1)	33.5 (32.1 – 34.9)	25.5 (24.3 – 26.7)

**Figure 3:**
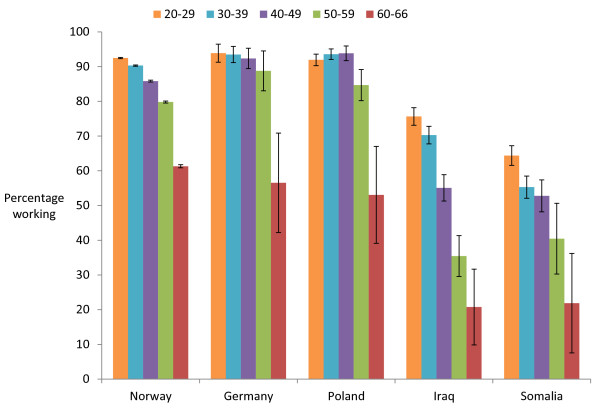
Percentage of EPHC patients working (95% CI), by country and age group.

**Figure 4:**
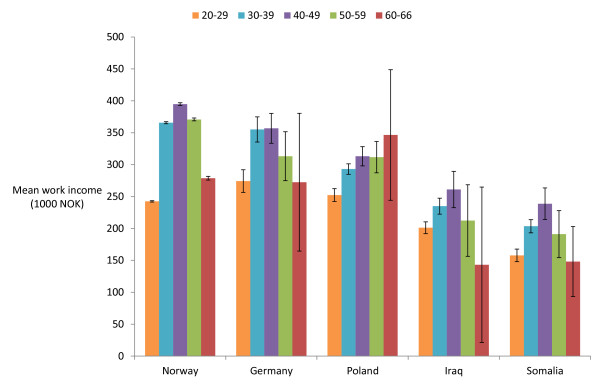
Mean annual income of EPHC patients working (95% CI), by country and age group.

Germans and Poles had employment rates quite similar to that of native Norwegians, while Iraqis, and especially Somalis, had lower employment rates (Figure 3). A similar pattern was found for the average work income of those actually working (Figure 4).

Immigrants from Poland and Germany had low EPHC contact rates, while Somalis and Iraqis had higher contact rates than native Norwegians (Table 2). Half of all contacts of Somalis and Iraqis were for non-specific pain, and they had relatively more of their contacts during night than other groups. Germans had the lowest need for interpreter, while Poles and Germans had the highest need for sickness certification. Laboratory tests were most frequently used for Somali patients.

**Table 2:** **Contact rates for different diagnoses, use of EPHC service at night, need for interpreter, long consultation, laboratory examination, and sickness certification **^**1 **^

	**Norway**	**Poland**	**Germany**	**Somalia**	**Iraq**
**Number of inhabitants (2008)**	4 110 812	48 826	36 700	24 232	25 238
**Number of contacts (any type)**	1 129 631	5 018	2 484	7 978	8 935
**Percentage contacts at night (0 am – 8 am)**	10.4 (10.3 – 10.5)	10.3 (9.5 – 11.1)	8.2 (7.1 – 9.3)	14.7 (13.9 – 15.5)	15.5 (14.8 – 16.3)
**Number of consultations (face-to-face with doctor)**	832 505	4 555	2 051	7 338	8 116
***Contact rates (per 100 inhabitants):***					
**All diagnoses**	27.4 (27.4 – 27.5)	11.9 (10.9 – 13.0)	7.0 (5.8 – 8.2)	31.8 (30.5 – 33.1)	33.6 (32.3 – 34.5)
**Non-specific pain**	5.9 (5.9 – 5.9)	3.7 (3.6 – 3.9)	1.8 (1.6 – 1.9)	16.8 (16.3 – 17.2)	15.7 (15.2 – 16.1)
**Injury**	2.7 (2.7 – 2.7)	1.1 (1.0 – 1.1)	0.8 (0.7 – 0.9)	1.5 (1.4 – 1.7)	1.7 (1.6 – 1.9)
**Psychiatric illness**	3.4 (3.4 – 3.4)	0.8 (0.7 – 0.9)	0.5 (0.5 – 0.6)	2.4 (2.2 – 2.6)	2.8 (2.6 – 3.0)
**Respiratory infections**	3.6 (3.6 – 3.6)	1.7 (1.6 – 1.8)	0.9 (0.8 – 1.0)	4.9 (4.6 – 5.1)	4.8 (4.6 – 5.1)
**Gastroenteritis**	0.2 (0.2 – 0.3)	0.1 (0.1 – 0.2)	0.1 (0.1 – 0.1)	0.7 (0.6 – 0.8)	0.6 (0.5 – 0.7)
***Rates of services (per100 consultations):***					
**Use of interpreter**	0.2 (0.2 – 0.2)	12.6 (11.6 – 13.6)	2.6 (1.9 – 3.3)	16.0 (15.2 – 16.8)	15.7 (14.9 – 16.5)
**Long consultation (> 20 minutes)**	37.8 (37.7 – 37.9)	40.3 (38.9 – 41.7)	39.8 (37.7 – 41.9)	41.0 (39.9 – 42.1)	33.9 (32.9 – 34.9)
**Use of laboratory**	33.8 (33.7 – 33.9)	37.2 (35.8 – 38.6)	33.2 (31.2 – 35.2)	51.1 (50.0 – 52.2)	43.5 (42.4 – 44.6)
**Sickness certification**	7.8 (7.7 – 7.9)	17.6 (16.5 – 18.7)	13.6 (12.1 –15.1)	8.5 (7.9 – 9.1)	7.6 (7.0 – 8.2)

^1^Rates and percentages are adjusted for age and presented with 95% CI.

The multivariate analyses confirmed that non-specific pain was more common among immigrants from Somalia and Iraq (Table 3). Work immigrants were most exposed to injuries and psychiatric illness, but psychiatric illness was also more common among asylum seekers than among family reunion immigrants. In addition, psychiatric illness increased with increasing length of stay in Norway. Work immigrants suffered less from respiratory and gastrointestinal infections, while immigrants from Somalia, Iraq, and Germany had more gastroenteritis.

**Table 3:** **Odds ratio (95% CI) for receiving different diagnoses **^**1 **^

	**Non-specific pain**	**Injury**	**Psychiatric illness**	**Respiratory infections**	**Gastroenteritis**
***Country of origin:***					
**Poland**	1.13 (0.89 – 1.43)	0.64 (0.49 – 0.84)	1.05 (0.68 – 1.62)	1.23 (1.01 – 1.51)	1.06 (0.62 – 1.82)
**Germany**	0.84 (0.64 – 1.10)	0.81 (0.60 – 1.07)	0.90 (0.56 – 1.46)	1.01 (0.81 – 1.28)	1.81 (1.02 – 3.21)
**Somalia**	2.03 (1.70 – 2.43)	0.41 (0.32 – 0.54)	0.85 (0.60 – 1.20)	0.88 (0.73 – 1.05)	2.01 (1.30 – 3.08)
**Iraq**	2.10 (1.77 – 2.50)	0.49 (0.38 – 0.63)	0.82 (0.58 – 1.15)	0.84 (0.70 – 1.00)	1.58 (1.02 – 2.44)
**Norway**	Ref.	Ref.	Ref.	Ref.	Ref.
***Reason for immigration:***					
**Protection**	1.16 (1.03 – 1.31)	0.92 (0.77 – 1.10)	1.43 (1.10 – 1.85)	1.17 (1.03 – 1.32)	0.98 (0.72 – 1.33)
**Work**	1.46 (1.19 – 1.79)	1.51 (1.25 – 1.82)	1.93 (1.30 – 2.85)	0.73 (0.62 – 0.86)	0.56 (0.34 – 0.90)
**Family reunion**	Ref.	Ref.	Ref.	Ref.	Ref.
***Length of stay:***					
**0 – 2 years**	0.97 (0.78 – 1.21)	1.27 (0.95 – 1.68)	0.26 (0.17 – 0.40)	1.02 (0.83 – 1.27)	1.28 (0.75 – 2.19)
**3 – 5 years**	0.95 (0.77 – 1.18)	1.33 (1.00 – 1.78)	0.55 (0.37 – 0.81)	1.08 (0.87 – 1.33)	0.63 (0.36 – 1.12)
**6 – 10 years**	1.09 (0.92 – 1.29)	1.26 (0.99 – 1.60)	0.78 (0.57 – 1.06)	1.15 (0.97 – 1.36)	0.88 (0.58 – 1.36)
**11 – 18 years**	Ref.	Ref.	Ref.	Ref.	Ref.

^1^Multivariate logistic regression analyses, adjusted for age, gender, centrality, Norwegian citizenship, work income, enlistment with a RGP, and number of EPHC contacts.

The multivariate analyses also confirmed that Somalis and Iraqis more often contacted the EPHC service at night (Table 4). Use of interpreter was mostly associated with country of origin and length of stay. All immigrant groups had an increased need for long consultations, while laboratory tests were most often used for Somalis and Iraqis. Being a work immigrant was the strongest predictor for receiving a sickness certificate, but immigrants from all countries, except Germany, were more often given a sickness certificate.

**Table 4:** **Odds ratio (95% CI) for contacting the EPHC service at night, need for interpreter, long consultation, laboratory examination, or sickness certification **^**1 **^

	**Night (0 am – 8 am)**	**Interpreter**	**Long consultation (> 20 min.)**	**Laboratory**	**Sickness certification**
***Country of origin:***					
**Poland**	0.92 (0.72 – 1.17)	38.29 (28.35 – 51.71)	1.33 (1.12 – 1.57)	1.41 (1.20 – 1.66)	1.96 (1.56 – 2.45)
**Germany**	0.74 (0.56 – 0.98)	9.07 (6.09 – 13.51)	1.45 (1.20 – 1.75)	1.24 (1.03 – 1.50)	1.16 (0.90 – 1.49)
**Somalia**	1.64 (1.37 – 1.96)	50.08 (38.22 – 65.62)	2.11 (1.83 – 2.43)	2.36 (2.05 – 2.72)	1.92 (1.58 – 2.34)
**Iraq**	1.67 (1.40 – 1.99)	60.32 (46.51 – 78.22)	1.50 (1.31 – 1.73)	1.81 (1.57 – 2.07)	1.90 (1.57 – 2.30)
**Norway**	Ref.	Ref.	Ref.	Ref.	Ref.
***Reason for immigration:***					
**Protection**	0.99 (0.88 – 1.13)	0.78 (0.67 – 0.90)	1.06 (0.96 – 1.17)	0.99 (0.90 – 1.09)	1.54 (1.33 – 1.77)
**Work**	1.11 (0.89 – 1.37)	1.56 (1.27 – 1.93)	1.13 (0.98 – 1.30)	0.70 (0.61 – 0.80)	3.47 (2.88 – 4.18)
**Family reunion**	Ref.	Ref.	Ref.	Ref.	Ref.
***Length of stay:***					
**0 – 2 years**	0.76 (0.60 – 0.94)	5.45 (4.01 – 7.41)	0.77 (0.65 – 0.92)	1.09 (0.92 – 1.29)	0.79 (0.63 – 0.99)
**3 – 5 years**	0.84 (0.67 – 1.04)	3.14 (2.31 – 4.26)	0.72 (0.61 – 0.86)	0.88 (0.74 – 1.04)	0.74 (0.58 – 0.93)
**6 – 10 years**	0.89 (0.75 – 1.06)	1.99 (1.54 – 2.56)	0.84 (0.73 – 0.96)	0.98 (0.85 – 1.12)	0.98 (0.82 – 1.17)
**11 – 18 years**	Ref.	Ref.	Ref.	Ref.	Ref.

^1^Multivariate logistic regression analyses, adjusted for age, gender, centrality, Norwegian citizenship, work income, enlistment with a RGP, and number of EPHC contacts.

## Discussion

On average, immigrants used the emergency services less than native Norwegians. This is in contrast to what has been described in surveys, where immigrants report greater use of emergency services than native Norwegians [6,24]. In a large survey conducted by Statistics Norway 2005 - 2006, immigrants reported 0.6 visits per year to the emergency services, native Norwegians 0.4. Compared with our study it seems that native Norwegians estimated their contact rate more correctly than immigrants [6]. People participating in surveys may not be representative of the whole population, and recall bias may affect their responses.

It should be noted, however, that the youngest children of immigrants used EPHC more often than native Norwegian children. Most of these children are second generation immigrants born in Norway, and the high contact rate probably reflects that their parents are insecure and lack support from older generations.

There were differences between immigrants from different parts of the world. Immigrants from Asia, Africa, and Latin America made most use of the emergency services, while immigrants from Western Europe had the lowest contact rate. Similar differences were found in the survey by Statistics Norway [6], and have also been reported from other European countries [18,25].

### Country of origin

The four immigrant nationalities examined in this study have some distinct features. Germans and Poles come to Norway for work. They have high employment rates, earn well, and have low rates of contact with the EPHC. They are typical representatives of the healthy migrant effect or healthy worker effect [2,3,26].

Iraqis and Somalis come to Norway for protection. They have lower employment rates and lower income, indicating low socio-economic status. In this respect Somalis seem to be most disadvantaged. Both Iraqis and Somalis have high EPHC contact rates.

The Germans have stayed in Norway for the longest time and are more geographically dispersed than other immigrants. They are older and have a gender distribution close to that of native Norwegians. Culturally, Germans are not very different from Norwegians, and they are probably the best integrated group. The Poles are mostly young males with a short length of stay in Norway. They are less geographically dispersed, and are almost as dependent on interpreters as Iraqis and Somalis.

Iraqis and Somalis are enlisted with a RGP to the same extent as native Norwegians, while 7 - 8% of Germans and Poles do not have a RGP. In Norway short term work immigrants are not entitled to a RGP.

When comparing EPHC contact rates between countries it should be noted that Norway has a high contact rate compared with some other countries with available data, e.g. almost twice the rate reported from Poland [27]. In our study Polish immigrants had a contact rate that was less than half of that of native Norwegians, indicating that they are selected by good health. This was even more evident for German immigrants, whose contact rate was only 26% of that of native Norwegians. Poles and Germans had low contact rates for all diagnostic groups examined, but were overrepresented among patients in need of sickness certification. This is probably explained by their high employment rates and the fact that short term work immigrants are not entitled to a RGP during the first months. Therefore, they have to get their sickness certificates at the EPHC service. However, when adjusted for other socio-demographic variables in a multivariate analysis, German immigrants did not differ significantly from native Norwegians, while the other immigrant groups were almost twice as prone to receiving a sickness certificate. It is possible that Germans have a higher work discipline than other immigrant groups, but they probably also have less manual work in which they can function despite minor illnesses.

Poland and Germany are not far from Norway, and it is possible that these immigrants go back to their home countries when they are sick, an example of the “unhealthy remigration effect” [13]. Immigrants who are used to direct access to specialists may be dissatisfied with the Norwegian health care system, based on RGPs and gate keeping, and remigrate for treatment [20,28,29]. Immigrants from Poland and Germany were much less likely to having acquired Norwegian citizenship than immigrants from Somalia and Iraq, also an indication that Poles and Germans want to keep closer contact with their country of origin.

Both Iraqi and Somali immigrants had higher EPHC contact rates than native Norwegians. The contact patterns of these two immigrant groups were very similar. They both had higher contact rates for infectious diseases, but most notable were the high rates for non-specific pain. Furthermore, they had a disproportionally high percentage of their contacts during night.

In health surveys Somali and Iraqi immigrants tend to place themselves in opposite ends of the scale [6,11,12]. Iraqis report much distress while Somalis are little bothered. This difference was not reflected at the EPHC service. Despite much better self-rated health we found that Somali immigrants used the EPHC services to a similar degree as Iraqi immigrants, a finding also noted in Denmark [30]. A possible explanation may be that Somali immigrants underreport health problems in surveys.

High contact rates at night and a lot of undiagnosed pain raise the suspicion that mental distress may be the real problem. Linguistic and cultural barriers create communication problems and an interpreter is seldom present at EPHC consultations. In some cultures it is uncommon or even shameful and taboo for patients to bring psychiatric problems to the doctor [31,32]. However, when uprooted from their familiar home country these immigrants have few other options than contacting the health care system. Instead of communicating anxiety or depression the patient may signalize severe pain while the doctor struggles to understand what is going on. Despite extensive use of laboratory tests and long consultations one is often left with a non-specific diagnosis of headache, back pain, or abdominal pain.

### Reason for immigration

Poles and Germans are typical examples of work immigrants, and as such benefited by both a healthy migrant effect and a healthy worker effect [2,3,26]. However, when they bring their families to Norway, it does not follow that their family members are also healthier than the average Pole or German.

To a certain extent contact rates at EPHC services reflected the reason for immigration. Work immigrants were less prone to infectious diseases, probably an effect of the healthy migrant or healthy worker effect. On the other hand they had more injuries and pain, and were more often in need of sickness certification. However, work immigrants were given a psychiatric diagnosis nearly twice as often as family reunion immigrants. Work immigrants were more often assisted by an interpreter, possibly enabling them to communicate more precisely with the doctor. It is also possible that work immigrants experience more stress than others, e.g. Polish immigrants who accept very long work hours and poor housing conditions.

Asylum seekers differed less from family reunion immigrants, but they were also more often in need of sickness certification. Probably, asylum seekers have higher employment rates themselves than their family members. Asylum seekers were also more often given a psychiatric diagnosis, but were less often assisted by an interpreter.

### Length of stay

As could be expected, the need for having an interpreter present at the consultation was reduced with the immigrant’s length of stay in Norway. Otherwise, there were few clear trends over time, with one notable exception. With increasing length of stay there was an increasing chance of receiving a psychiatric diagnosis.

There are several possible explanations for this trend towards increasing psychiatric illness with time. Improved linguistic skills and adaptation to Norwegian help seeking behaviour make it easier to explain the problem to the doctor on call. Instead of non-specific psychosomatic pain a more specific diagnosis of depression or anxiety may be given. However, if a diagnostic shift was the reason for more frequent use of psychiatric diagnoses, one would expect reduced frequency of non-specific pain diagnoses, and this is not the case. Therefore, other explanations must also be considered.

It is well known that immigrants may experience mental stress because of difficulties in their new country [11]. Poor knowledge of language and culture may cause isolation and loneliness. Many immigrants face discrimination and have poor socio-economic status compared with native Norwegians. In Norway dark winters and a harsh climate may also contribute to mental health problems. This theory is in accordance with the findings of another Norwegian study in which it was concluded that post-migration factors in the host country are most important for psychological distress among immigrants [14]. It is also in accordance with the theory that the healthy migrant effect may wear off with time [4,5].

### Limitations

This is a study of established immigrants’ use of the EPHC. Foreign tourists, illegal residents and many short term visitors and asylum seekers are not included in the material. Also, Norwegians and immigrants who could not remember their ID-number are excluded. Among these, children are obviously overrepresented. Probably, many parents have difficulties remembering their children’s ID-numbers.

We don’t know how many of the missing ID-numbers are persons who don’t have such numbers (and therefore would be excluded anyhow) and how many are immigrants or native Norwegians who could have been included. We have no reason to believe that established immigrants and native Norwegians differ in their ability to remember their ID-number. Therefore, comparisons between groups are probably valid. However, the contact rates found here will be a little lower than the real rates.

Short term visitors who had a temporary D-number in 2008 were only included in our study if they had been given an ID-number before 2010. This may have introduced a bias in the material, since the percentage of short term visitors may vary with country of origin.

Work income only applies to the individual worker, and not to his family. Thus, we have little information about the socio-economic status of those not working. Reasons for immigration have only been recorded by Statistics Norway since 1990. Therefore, we had to exclude immigrants from the multivariate analyses if they immigrated to Norway before 1990.

## Conclusion

Most immigrants use EPHC services less than native Norwegians, but they take their youngest children to the EPHC more often than native Norwegians. Adjusted for a series of socio-demographic and socio-economic variables we found that immigrants’ use of EPHC services varies with their country of origin, their reason for immigration, and with their length of stay in Norway. Work immigrants from Germany and Poland use EPHC considerably less, while asylum seekers from Somalia and Iraq use these services more than native Norwegians.

## Abbreviations

CI: Confidence interval; EPHC: Emergency primary health care; HELFO: Norwegian Health Economics Administration; ICPC: International classification of primary care; NOK: Norwegian kroner; RGP: Regular general practitioner.

## Competing interests

The authors declare that they have no competing interests.

## Authors’ contribution

ED conceived the main project “Immigrants’ health in Norway” and obtained the data. HS designed the present study, analyzed the data, and drafted the manuscript. All authors participated in the interpretation of the data, revising the manuscript, and approving the final version.

## Pre-publication history

The pre-publication history for this paper can be accessed here:

http://www.biomedcentral.com/1472-6963/12/308/prepub
